# Supplementary material for: SELENOP rs3877899 Variant Affects the Risk of Developing Advanced Stages of Retinopathy of Prematurity (ROP)
Source: Int J Mol Sci. 2023 Apr 20;24(8):7570. doi: 10.3390/ijms24087570 (PMC10145309; doi:10.3390/ijms24087570)
Supplement: Supplementary file 1 [file ijms-24-07570-s001.zip › ijms-2308718-supplementary.pdf]

Supplemental Table S1. Distribution of gene polymorphisms according to ROP (retinopathy of prematurity) presence, severity, and treatment outcome.

Genotype	Incidence and Outcome of ROP			Dose of allele β, p for trend I/II/III	ROP Requiring Treatment		Dose of allele p IIIa vs. IIIb
	I No ROP N=52	II ROP Not Requiring Treatment N=55	III ROP Requiring Treatment N=66		IIIa ROP Treated Successfully N=47	IIIb ROP Treated Unsuccessfully N=19	
SELENOP rs3877899G>A							
GG	32 (61.5)	32 (58.2)	32 (48.5)		24 (51.1)	8 (42.1)	
GA	19 (36.5)	18 (32.7)	26 (39.4)		19 (40.4)	7 (36.8)	
AA	1 (1.9)	5 (9.1)	8 (12.1)		4 (8.5)	4 (21.1)	
MAF	**0.202**[a,b]	0.255	**0.318**[a]	**0.150; <0.050**	0.287	**0.395**[b]	0.517
SELENOP rs7579G>A							
GG	22 (43.1)	29 (52.7)	38 (57.6)		23 (48.9)	15 (78.9)	
GA	25 (49.0)	23 (41.8)	25 (37.9)		22 (46.8)	3 (15.8)	
AA	4 (7.8)	3 (5.5)	3 (4.5)		2 (4.3)	1 (5.3)	
MAF	**0.324**[c]	0.264	0.235	-0.119; 0.121	0.277	**0.132**[c]	0.069
SELENOS rs34713741C>T							
CC	29 (56.9)	29 (52.7)	35 (53.0)		27 (57.4)	8 (42.1)	
CT	14 (27.5)	25 (45.5)	27 (40.9)		17 (36.2)	10 (52.6)	
TT	8 (15.7)	1 (1.8)	4 (6.1)		3 (6.4)	1 (5.3)	
MAF	0.294	0.245	0.265	-0.033; 0.659	0.245	0.316	0.398
GPX4 rs713041C>T							
CC	23 (44.2)	21 (38.2)	25 (37.9)		15 (31.9)	10 (52.6)	
CT	18 (34.6)	26 (47.3)	30 (45.5)		24 (51.1)	6 (31.6)	
TT	11 (21.2)	8 (14.5)	11 (16.7)		8 (17.0)	3 (15.8)	
MAF	0.385	0.382	0.394	0.011; 0.883	0.426	0.316	0.261

Statistical analysis: a, b -*SELENOP* rs3877899: a: I vs. III: A allele: OR=1.8 (95%C.I.: 1.0-3.4), *p*=0.045; AA genotype (recessive): OR=7.0 (95%C.I.: 1.0-58.2), *p*=0.038; b: I vs. IIIb: A allele: OR=2.6 (95%C.I.: 1.1-5.8), *p*=0.019; AA genotype (recessive): OR=13.6 (95%C.I.: 1.4-131.1), *p*=0.005; c: *SELENOP* rs7579A I vs. IIIb: A allele: OR=0.32 (95%C.I.:0.11-0.89), *p*=0.023; AA+GA genotype (dominant): OR=0.20 (95%C.I.: 0.06-0.70), *p*=0.008.

Supplemental Table S2. Distribution of gene polymorphisms according to the presence of complications of prematurity: RDS, IVH, PDA, NEC, BPD, extremely low gestational age, and extremely low birth weight.

Genotype	No RDS N=81	RDS N=101	No IVH N=61	IVH N=121	No PDA N=128	PDA N=54	No NEC N=134	NEC N=47	No BPD N=100	BPD N=82	≥28 wk. n=102	<28 wk. n=82	≥1000 g n=104	<1000 g n=80
SELENOP* rs3877899G>*A														
**GG**	40 (49.4)	61 (60.4)	31 (50.8)	70 (57.9)	75 (58.6)	26 (48.1)	76 (56.7)	24 (51.1)	54 (54.0)	47 (57.3)	64 (62.7)	39 (47.6)	60 (57.7)	43 (53.8)
**GA**	33 (40.7)	34 (33.7)	25 (41.0)	42 (34.7)	41 (32.0)	26 (48.1)	51 (38.1)	16 (34.0)	40 (40.0)	27 (32.9)	**33 (32.4)**	**34 (41.5)**	38 (36.5)	29 (36.3)
**AA**	8 (9.9)	6 (5.9)	5 (8.2)	9 (7.4)	12 (9.4)	2 (3.7)	7 (5.2)	7 (14.9)	6 (6.0)	8 (9.8)	**5 (4.9)**	**9 (11.0)**	6 (5.8)	8 (10.0)
**MAF**	0.302	0.228	0.287	0.248	0.254	0.278	0.243	0.319	0.260	0.262	**0.211**[a]	**0.317**[a]	0.240	0.281
SELENOP* rs7579G>*A														
**GG**	40 (49.4)	61 (60.4)	31 (50.8)	70 (57.9)	68 (53.1)	26 (49.1)	66 (49.3)	28 (60.9)	46 (46.0)	48 (59.3)	48 (47.1)	46 (56.8)	52 (50.5)	42 (52.5)
**GA**	33 (40.7)	34 (33.7)	25 (41.0)	42 (34.7)	53 (41.4)	24 (45.3)	60 (44.8)	16 (34.8)	48 (48.0)	29 (35.8)	46 (45.1)	32 (39.5)	46 (44.7)	32 (40.0)
**AA**	8 (9.9)	6 (5.9)	5 (8.2)	9 (7.4)	7 (5.5)	3 (5.7)	8 (6.0)	2 (4.3)	6 (6.0)	4 (4.9)	8 (7.8)	3 (3.7)	5 (4.9)	6 (7.5)
**MAF**	0.244	0.287	0.275	0.264	0.262	0.283	0.284	0.217	0.300	0.228	0.304	0.235	0.272	0.275
SELENOS* rs34713741C>*T														
**CC**	40 (50.0)	57 (56.4)	32 (53.3)	65 (53.7)	66 (51.6)	31 (58.5)	71 (53.0)	26 (56.5)	48 (48.0)	49 (60.5)	53 (52.0)	44 (54.3)	51 (49.5)	46 (57.5)
**CT**	37 (46.3)	34 (33.7)	24 (40.0)	47 (38.8)	51 (39.8)	20 (37.7)	53 (39.6)	17 (37.0)	42 (42.0)	29 (35.8)	37 (36.3)	34 (42.0)	42 (40.8)	29 (36.3)
**TT**	3 (3.8)	10 (9.9)	4 (6.7)	9 (7.4)	11 (8.6)	2 (3.8)	10 (7.5)	3 (6.5)	10 (10.0)	3 (3.7)	12 (11.8)	3 (3.7)	10 (9.7)	5 (6.3)
**MAF**	0.269	0.267	0.267	0.269	0.285	0.226	0.272	0.250	0.310	0.216	0.299	0.247	0.301	0.244
GPX4* rs713041C>*T														
**CC**	30 (37.0)	42 (41.6)	27 (44.3)	45 (37.2)	53 (41.4)	19 (35.2)	55 (41.0)	16 (34.0)	42 (42.0)	30 (36.6)	43 (42.2)	31 (37.8)	43 (41.3)	*31 (38.8)*
**CT**	36 (44.4)	44 (43.6)	25 (41.0)	55 (45.5)	55 (43.0)	25 (46.3)	59 (44.0)	21 (44.7)	44 (44.0)	36 (43.9)	43 (42.2)	37 (45.1)	47 (45.2)	*33 (41.3)*
**TT**	15 (18.5)	15(14.9)	9 (14.8)	21 (17.4)	20 (15.6)	10 (18.5)	20 (14.9)	10 (21.3)	14 (14.0)	16 (19.5)	16 (15.7)	14 (17.1)	14 (13.5)	*16 (20.0)*
**MAF**	0.407	0.366	0.352	0.401	0.371	0.417	0.369	0.436	0.360	0.415	0.368	0.396	0.361	*0.406*

Abbreviations and symbols: BPD-bronchopulmonary dysplasia; IVH-intraventricular hemorrhage; PDA-patent ductus arteriosus; RDS-respiratory distress syndrome; ROP-retinopathy of prematurity, NEC-necrotizing enterocolitis. Statistical analysis: a - *SELENOP* rs3877899A allele: OR=1.7 (95%C.I. 1.1-2.8), *p*=0.02; carriers of the rs3877899A allele (AA+GA genotypes): OR=1.9 (95%C.I.: 1.0-3.4), *p*=0.04.
